# Patterns of Internet Addiction in an Italian sample: 100% of the sample experience Nomophobia

**DOI:** 10.1192/j.eurpsy.2024.1139

**Published:** 2024-08-27

**Authors:** F. Marcolini, D. De Ronchi, A. R. Atti

**Affiliations:** Department of Biomedical and Neuromotor Sciences, University of Bologna, Bologna, Italy

## Abstract

**Introduction:**

Internet Addiction Disorder, a concept introduced for the first time by Ivan Goldberg in 1995, is one of the most recently identified forms of addiction, but already considered a real psychosocial phenomenon, capable of having a profound impact on different aspects of social and psychological life of individuals. One of its most recently identified manifestations is Nomophobia, a neologism formed from the combination of terms such as “no mobile”, “phone” and “phobia”, which can be understood as the fear of feeling disconnected. It is today considered a situational phobia, characteristic of contemporary times. The most common symptoms include excessive cell phone use and constant anxiety at the thought of losing the internet connection. Others are, for example, “Ringxiety”, ringing anxiety, or the “phantom vibration syndrome”.

**Objectives:**

This study aims to examine the spread of Nomophobia in the Italian population, evaluating psychopathological correlations that can explain its diffusion.

**Methods:**

Between January and May 2023, an anonymous online questionnaire was randomly sent to the general population. Alongside with tests to evaluate psycho-social features, the instrument used to study Nomophobia was the *Nomophobia Questionnaire* (NMP-Q) (Yildrim *et al.* Comput Hum Behav. 2015; 49:130–7), in its Italian version (Adawi *et al.* JMIR MHealth UHealth. 2018;6:e24).

**Results:**

The sample consists of 308 people (189 F, 119 M), with an average age of 32 years (*sd* 14). In our sample, 100% of the subjects tested positive for Nomophobia. Values indicating a state of severe Nomophobia are found in 12.3% of the sample (F 15.9%, M 6.7%). The young population, between 18 and 25 years old, represents 54% of the affected population, but more than 60% of severe cases (95% confidence interval 50-65%). The severe cases correlate positively (p<0,05) with findings of high impulsiveness. There are no other studies that investigate the psychopathological correlates of Nomophobia among Italians.

**Conclusions:**

Despite possible *biases,* the data obtained are an alarming sign of the spread of internet addiction that characterizes our times, of which the excessive use of cell phones in the form of Nomophobia is an expression. Despite their now undisputed usefulness, mobile devices are capable of causing the onset of serious health problems, starting from exposure to radiation capable of causing dermatitis, tumors, and infertility. Furthermore, they dramatically interfere with driving safety, becoming a major cause of road accidents. Considering these consequences, it appears to be extremely important to characterize the phenomenon, as well as its psychosocial determinants, in order to proceed with its better definition and prevention.

**Disclosure of Interest:**

None Declared

